# Supplementary material for: Question-based computational language approach outperforms rating scales in quantifying emotional states
Source: Commun Psychol. 2024 May 23;2:45. doi: 10.1038/s44271-024-00097-2 (PMC11332055; doi:10.1038/s44271-024-00097-2)
Supplement: Supplementary file 2 — Reporting Summary [file 44271_2024_97_MOESM2_ESM.pdf]

nature portfolio

Corresponding author(s): Sverker Sikström

Last updated by author(s): 2024.03.26

# Reporting Summary

Nature Portfolio wishes to improve the reproducibility of the work that we publish. This form provides structure for consistency and transparency in reporting. For further information on Nature Portfolio policies, see our Editorial Policies and the Editorial Policy Checklist.

## Statistics

For all statistical analyses, confirm that the following items are present in the figure legend, table legend, main text, or Methods section.

| n/a | Confirmed | |
|---|---|---|
| ☐ | ☒ | The exact sample size (*n*) for each experimental group/condition, given as a discrete number and unit of measurement |
| ☐ | ☒ | A statement on whether measurements were taken from distinct samples or whether the same sample was measured repeatedly |
| ☐ | ☒ | The statistical test(s) used AND whether they are one- or two-sided<br>*Only common tests should be described solely by name; describe more complex techniques in the Methods section.* |
| ☒ | ☐ | A description of all covariates tested |
| ☒ | ☐ | A description of any assumptions or corrections, such as tests of normality and adjustment for multiple comparisons |
| ☐ | ☒ | A full description of the statistical parameters including central tendency (e.g. means) or other basic estimates (e.g. regression coefficient) AND variation (e.g. standard deviation) or associated estimates of uncertainty (e.g. confidence intervals) |
| ☐ | ☒ | For null hypothesis testing, the test statistic (e.g. $F$, $t$, $r$) with confidence intervals, effect sizes, degrees of freedom and $P$ value noted<br>*Give P values as exact values whenever suitable.* |
| ☒ | ☐ | For Bayesian analysis, information on the choice of priors and Markov chain Monte Carlo settings |
| ☒ | ☐ | For hierarchical and complex designs, identification of the appropriate level for tests and full reporting of outcomes |
| ☐ | ☒ | Estimates of effect sizes (e.g. Cohen's $d$, Pearson's $r$), indicating how they were calculated |

*Our web collection on statistics for biologists contains articles on many of the points above.*

## Software and code

Policy information about availability of computer code

| | |
|---|---|
| Data collection | Participants were directed from Prolific, a platform for recruiting participants for research studies online, to a Qualtrics questionnaire, in which their responses were collected. |
| Data analysis | The corresponding author can provide the custom computer code, upon request, in a manner that enables readers to replicate the published results. |

For manuscripts utilizing custom algorithms or software that are central to the research but not yet described in published literature, software must be made available to editors and reviewers. We strongly encourage code deposition in a community repository (e.g. GitHub). See the Nature Portfolio guidelines for submitting code & software for further information.

## Data

Policy information about availability of data

All manuscripts must include a data availability statement. This statement should provide the following information, where applicable:

- Accession codes, unique identifiers, or web links for publicly available datasets
- A description of any restrictions on data availability
- For clinical datasets or third party data, please ensure that the statement adheres to our policy

The design, hypotheses, and analysis plan for this manuscript were preregistered in the Open Science Framework (OSF) prior to the completion of the study. The original Qualtrics surveys, supplementary materials, and anonymized participant data have been made publicly available at https://osf.io/6ydfj.

## Human research participants

Policy information about studies involving human research participants and Sex and Gender in Research.

| | |
|---|---|
| Reporting on sex and gender | Participants were asked to indicate their gender as part of the demographic data collection. |
| Population characteristics | Participants were asked to indicate their country of birth and level of completed education as part of the demographic data collection. |
| Recruitment | Participants were recruited through Prolific, an online recruitment platform for data collection in the behavioral sciences. he same platform was used to recruit healthcare professionals by screening participants based on their indication of having a professional occupation within the healthcare system as either; a doctor, emergency medical employee, nurse, paramedic, pharmacist, psychologist, or social worker. Participants were compensated £2 in Phase 1 and £1.5 in Phase 2 for participating in the study. As such, participants' responses may have been affected by the desire to earn money rather than a genuine interest in contributing to scientific inquiry. |
| Ethics oversight | The study received ethical approval from the Regional Ethics Board in Lund and adhered to Swedish laws (Dnr 2021-04627). Informed consent was obtained by participants prior to the start of the experiment. |

Note that full information on the approval of the study protocol must also be provided in the manuscript.

# Field-specific reporting

Please select the one below that is the best fit for your research. If you are not sure, read the appropriate sections before making your selection.

☐ Life sciences ☒ Behavioural & social sciences ☐ Ecological, evolutionary & environmental sciences

For a reference copy of the document with all sections, see nature.com/documents/nr-reporting-summary-flat.pdf

# Behavioural & social sciences study design

All studies must disclose on these points even when the disclosure is negative.

| | |
|---|---|
| Study description | The study demonstrates that descriptive word responses analyzed using NLP show higher accuracy in categorizing emotional states compared to traditional rating scales. |
| Research sample | The inclusion criteria were 18 years of age or older and being a native English language speaker. The study had two phases with different sets of participants but with the same inclusion criteria. Thus, the final sample from both phases consisted of 731 participants (female = 428; male = 281; other = 22) with an age range of 18-79 years (M = 31.97, SD = 12.71). Although the majority of participants came from either USA (N = 350) or UK (N = 218), there were some participants that currently reside in other coutnries (N = 163). This is thus a representative sample of native english language speakers. |
| Sampling strategy | The data used here were collected by convenience sampling. |
| Data collection | Participants were recruited through Prolific, an online recruitment platform for data collection in the behavioral sciences. |
| Timing | The data was collected in April, 2022 |
| Data exclusions | A total of 350 participants completed the study in Phase 1, and 53 participants were removed due to failure to correctly answer the control questions, leaving a final sample size of 297. A total of 465 participants completed the study in Phase 2, 34 of whom were healthcare professionals recruited through additional screening. A total of 31 participants were removed as they did not respond correctly to the control questions, leaving a final sample size of 434. |
| Non-participation | No participants dropped out of the study. |
| Randomization | In Phase 1, the allocation of psychological constructs for participants to write about was done randomly, ensuring that each construct received an approximately equal number of responses. In Phase 2, participants were presented with the texts to read in a random order, ensuring that each text was read at least once, but no more than twice. |

# Reporting for specific materials, systems and methods

We require information from authors about some types of materials, experimental systems and methods used in many studies. Here, indicate whether each material, system or method listed is relevant to your study. If you are not sure if a list item applies to your research, read the appropriate section before selecting a response.

## Materials & experimental systems

| n/a | Involved in the study |
|---|---|
| ☒ | ☐ Antibodies |
| ☒ | ☐ Eukaryotic cell lines |
| ☒ | ☐ Palaeontology and archaeology |
| ☒ | ☐ Animals and other organisms |
| ☒ | ☐ Clinical data |
| ☒ | ☐ Dual use research of concern |

## Methods

| n/a | Involved in the study |
|---|---|
| ☒ | ☐ ChIP-seq |
| ☒ | ☐ Flow cytometry |
| ☒ | ☐ MRI-based neuroimaging |
